# Prevalence, Method Mix, and Determinants of Contraceptive Use Among Eligible Couples in an Urban Resettlement Colony of Delhi

**DOI:** 10.7759/cureus.102715

**Published:** 2026-01-31

**Authors:** Suresh Bangla, Sanjay Rai, Kapil Yadav, Baridalyne Nongkynrih

**Affiliations:** 1 Centre for Community Medicine, All India Institute of Medical Sciences, New Delhi, New Delhi, IND

**Keywords:** contraception, contraceptive prevalence, dmpa, family planning, female reproductive health, india, information on contraception, method mix, predictors, urban resettlement colony

## Abstract

Background

Although contraceptive prevalence in Delhi is relatively high, urban resettlement colonies may exhibit distinct patterns of contraceptive utilisation and method choice that are not captured in aggregate estimates. Understanding the prevalence, method mix, and factors associated with contraceptive use in such settings is essential for strengthening urban family planning services.

Objectives

To assess the prevalence, method mix, and predictors of current contraceptive use among eligible couples residing in an urban resettlement colony of Delhi.

Methods

A community-based cross-sectional study was conducted among 306 eligible couples, selected by simple random sampling from households in an urban resettlement colony in South-East Delhi. Data were collected using a pre-tested questionnaire adapted from the National Family Health Survey. Descriptive statistics were used to estimate awareness, ever use, current use, and contraceptive method mix. Multivariable logistic regression analysis was performed to identify factors independently associated with current contraceptive use, and adjusted odds ratios (AORs) with 95% confidence intervals (CI) were reported.

Results

Among 306 eligible couples, awareness of at least one contraceptive method was reported by 300 (98.0%). Ever use of any contraceptive method was reported by 207 (67.6%), while 179 (58.5%) were current users at the time of the survey. Among current users, condoms were the most commonly reported method, used by 73 (23.9%) participants, followed by female sterilisation in 56 (18.3%). Use of modern spacing methods was lower, with intrauterine devices reported by 18 (5.9%), oral contraceptive pills by 17 (5.6%), and injectable contraception by 14 (4.6%). Partner support among current users was reported by 171 (95.5%). Joint decision-making regarding contraceptive use was reported by 96 (53.6%) current users. On multivariable analysis, longer duration of marriage was positively associated with current contraceptive use (AOR = 1.14, 95% CI: 1.02-1.28). Having one or more male children showed a strong association (AOR = 3.17, 95% CI: 1.72-5.84), as did contact with Accredited Social Health Activist (ASHA) (AOR = 3.48, 95% CI: 1.79-6.77). Educational attainment demonstrated a graded association, with significantly higher odds among women with higher secondary education (AOR = 10.85, 95% CI: 3.21-36.69), secondary education (AOR = 6.64, 95% CI: 2.15-20.56), and primary education (AOR = 3.67, 95% CI: 1.16-11.62), compared to those with no schooling.

Conclusion

Contraceptive use among eligible couples in this urban resettlement colony varied by stage of marital and reproductive life and by engagement with community-based health services, despite high levels of awareness. The findings describe a contraceptive method mix dominated by condoms and highlight the association of contraceptive use with educational status, marital duration, child sex composition, and recent contact with frontline health workers. These results provide context-specific evidence on patterns and correlates of contraceptive use in an urban resettlement setting.

## Introduction

Contraceptive use is a cornerstone of reproductive health and a critical public health intervention for preventing unintended pregnancies, reducing unsafe abortions, and lowering maternal mortality [1]. Globally, an estimated 257 million women of reproductive age have an unmet need for modern contraception, with the majority residing in low- and middle-income countries [2]. In India, despite significant progress in family planning programmes over the past several decades, contraceptive prevalence remains suboptimal, and substantial disparities persist across geographic, socioeconomic, and demographic strata [3].

According to the National Family Health Survey-5 (NFHS-5), the contraceptive prevalence rate (CPR) among currently married women aged 15-49 years in India was 66.7%, with marked variation between urban (69.6%) and rural (65.2%) areas [4]. However, these aggregate figures mask considerable heterogeneity within urban populations, particularly among residents of slums and resettlement colonies, who face unique barriers to accessing reproductive health services [5]. Urban resettlement colonies, characterised by high population density, inadequate infrastructure, and limited access to quality healthcare, represent a vulnerable subpopulation that has received insufficient attention in family planning research [6].

Previous studies in urban India have documented contraceptive prevalence rates ranging from 45% to 70%, with condoms and female sterilisation being the most commonly used methods [7-9]. Evidence examining the full spectrum of contraceptive methods in urban resettlement colonies remains limited. This study aimed to assess the prevalence, method mix, and determinants of contraceptive use among eligible couples in an urban resettlement colony of Delhi.

## Materials and methods

Study design and setting

This was a community-based, cross-sectional study conducted in Dakshinpuri Extension, an urban resettlement colony located in South-East Delhi. Dakshinpuri Extension serves as the urban field practice area (UFPA) of the Centre for Community Medicine, All India Institute of Medical Sciences (AIIMS), New Delhi. The area has an estimated total population of approximately 35,618 individuals, with approximately 6,000 eligible couples (married women of reproductive age, 18-45 years, living with their husbands) [10].

The study was conducted between August 2025 and September 2025. The area is characterised by high population density, mixed socioeconomic status, and access to both public and private healthcare facilities. Primary healthcare services are provided through an Urban Primary Health Centre (UPHC) and supported by a network of Accredited Social Health Activists (ASHAs) and auxiliary nurse midwives (ANMs).

Study population and sampling

The study population comprised eligible couples, defined as married women aged 18-45 years currently living with their husbands and residing in the study area for at least six months. Women who were pregnant at the time of the survey or had undergone a hysterectomy were excluded.

For the selection of the households, a simple random sampling method was employed using the RAND function. A sampling frame was prepared using the Health Management Information System (HMIS) list maintained by the Centre for Community Medicine, which contains household-level data on all registered families.

Sample size calculation

The sample size was calculated using the formula for estimating a single proportion:

$N=Z^{2}pq/d^{2}$,

where: Z = 1.96 (for 95% confidence level), p = 0.764 (contraceptive prevalence rate in urban Delhi from NFHS-5) [4], q = 0.236, and d = 0.05 (absolute precision).

Substituting these values:

n = (1.96)² × 0.764 × (1 - 0.764)/(0.05)², n = 3.8416 × 0.764 × 0.236/0.0025, n = 0.6926/0.0025, n = 277.

To account for a 10% non-response rate, the sample size was inflated: n = 277 + 10% = 305. A total of 306 eligible couples were successfully enrolled and interviewed, exceeding the minimum required sample size.

Data collection

Data were collected through face-to-face interviews using a pre-tested, semi-structured questionnaire. The questionnaire was translated into Hindi and back-translated to ensure linguistic and cultural appropriateness. The questionnaire captured information on socio-demographic characteristics, reproductive history, awareness of contraceptive methods, ever and current use of contraception, method-specific details including type and source of method, partner support and decision-making, and recent contact with ASHA workers. The questionnaire used for data collection is provided in Appendix A and was adapted from the National Family Health Survey-5 and permission for the general usage is given [4].

Interviews were conducted by trained female investigators at the participants' homes to ensure privacy and confidentiality. Data were collected on paper-based forms and subsequently entered into a digital database.

Statistical analysis

Data were analysed using SPSS version 26.0 (IBM Corp., Armonk, NY, USA). Descriptive statistics were used to summarise socio-demographic characteristics, reproductive variables, awareness of contraceptive methods, and patterns of ever and current contraceptive use. Categorical variables were expressed as frequencies and percentages, while continuous variables were summarised using medians with interquartile ranges due to non-normal distribution.

Bivariate analysis was performed to examine the association between current contraceptive use and selected independent variables. The chi-square test was used for categorical variables, while the Wilcoxon rank-sum test was applied for continuous variables owing to non-parametric distribution.

Variables with a p-value of less than 0.25 in bivariate analysis were included in a multivariable logistic regression model to identify independent determinants of current contraceptive use. Adjusted odds ratios with 95% confidence intervals were estimated, and statistical significance was set at p < 0.05.

## Results

Socio-demographic characteristics

A total of 306 eligible couples were included in the analysis. Table 1 presents the socio-demographic and household characteristics of the study participants.

**Table 1:** Socio-demographic and household characteristics of eligible couples participating in the study (N = 306).

Variable	Category	n	%
Age group (years)	18–24	101	33.0
	25–29	90	29.4
	30–34	60	19.6
	35–39	35	11.4
	40–45	20	6.5
Educational attainment	No schooling	37	12.1
	Primary (1–5 years)	56	18.3
	Secondary (6–10 years)	99	32.4
	Higher secondary (11–12 years)	106	34.6
	Graduate and above	8	2.6
Occupation	Not employed	250	81.7
	Employed	56	18.3
Monthly household income (INR)	<10,000	8	2.6
	10,000–14,999	18	5.9
	15,000–19,999	30	9.8
	20,000–24,999	108	35.3
	25,000–29,999	30	9.8
	≥30,000	112	36.6
Religion	Hindu	272	88.9
	Muslim	30	9.8
	Others	4	1.3
Family type	Nuclear	236	77.1
	Joint	70	22.9
Number of living children	0	51	16.7
	1	88	28.8
	2	135	44.1
	≥3	32	10.5

The largest proportion of participants belonged to the 18-24 years age group, comprising 101 (33.0%) individuals, followed by those aged 25-29 years with 90 (29.4%). With respect to educational attainment, higher secondary education (11-12 years) was the most common level, reported by 106 (34.6%) participants, followed by secondary education (6-10 years) in 99 (32.4%). Only 8 (2.6%) participants were graduates or above, while 37 (12.1%) had no formal schooling.

The majority of participants were not engaged in paid employment, accounting for 250 (81.7%). Monthly household income was most commonly reported in the ≥30,000 INR category by 112 (36.6%) participants, followed by the 20,000-24,999 INR group with 108 (35.3%). Most participants belonged to the Hindu religion, reported by 272 (88.9%), and resided in nuclear families, reported by 236 (77.1%). Regarding fertility profile, the most frequently reported category was two living children, observed in 135 (44.1%) participants, followed by one child in 88 (28.8%). Table 2 presents descriptive statistics for key continuous variables.

**Table 2:** Descriptive statistics of key continuous variables among participants (N = 306). IQR: interquartile range.

Variable	Mean ± SD	Median (IQR)	Range
Age (years)	29.1 ± 7.0	28 (23–34)	18–45
Age at marriage (years)	21.8 ± 2.7	21 (21–22)	16–35
Duration of marriage (years)	7.3 ± 6.6	5 (2–10)	0–24
Monthly household income (INR)	25,680.8 ± 8790.9	24,000 (20,000–31,000)	14,000–50,000
Number of living children	1.46 ± 1.07	1 (1–2)	0–4
Number of male children	0.70 ± 0.71	1 (0–1)	0–3
Number of female children	0.91 ± 0.70	1 (0–1)	0–3
Age of youngest child (years)	1.79 ± 2.90	1 (0–2)	0–16

The mean age of the participants was 29.1 ± 7.0 years, with a median age of 28 years (IQR: 23-34). The mean age at marriage was 21.8 ± 2.7 years, and the median age at marriage was 21 years (IQR: 21-22). The mean duration of marriage was 7.3 ± 6.6 years, with a median duration of five years (IQR: 2-10). The mean monthly household income was INR 25,680.8 ± 8790.9, with a median income of INR 24,000 (IQR: 20,000-31,000). The mean number of living children was 1.46 ± 1.07, with a median of one child (IQR: 1-2).

Awareness and prevalence of contraceptive use

Table 3 presents the prevalence of awareness and use of contraceptive methods among eligible couples.

**Table 3:** Prevalence of awareness and use of contraceptive methods among eligible couples (N = 306).

Metric	Value (%)	95% CI
Ever heard of any contraceptive method	98.0	95.7–99.1
Ever used any contraceptive method	67.6	62.2–72.6
Currently using any contraceptive method	58.5	52.9–63.9

Among the 306 participants, awareness of at least one contraceptive method was reported by 300 (98.0%; 95% CI: 95.7-99.1). Ever use of any contraceptive method was reported by 207 (67.6%; 95% CI: 62.2-72.6). At the time of the survey, 179 (58.5%; 95% CI: 52.9-63.9) participants were currently using a contraceptive method. Table 4 presents method-specific awareness and contact with ASHA workers.

**Table 4:** Awareness of different contraceptive methods and contact with ASHA among eligible couples (N = 306). ASHA: Accredited Social Health Activists; OCP: oral contraceptive pills; DMPA: depot medroxyprogesterone acetate, IUCD: intrauterine contraceptive device; IUD: intrauterine device.

Section	Category	n	%
Overall awareness	Heard of any method	300	98.0
	Not heard/no response	6	2.0
Method-specific awareness	Female sterilisation	289	94.4
	Condoms	276	90.2
	IUD/IUCD	261	85.3
	Pills (OCP)	259	84.6
	Male sterilisation	172	56.2
	Withdrawal method	170	55.6
	Injectables (DMPA/Antara)	164	53.6
	Lactational amenorrhoea	158	51.6
	Emergency contraceptive pills	86	28.1
	Rhythm method	65	21.2
	Implants	30	9.8
Contact with ASHA	Yes	223	72.9
	No	83	27.1

Overall awareness of contraception was high in the study population, with 300 (98.0%) participants reporting that they had heard of at least one contraceptive method. Among method-specific awareness, female sterilisation was the most commonly known method, reported by 289 (94.4%) participants, followed by condoms in 276 (90.2%), intrauterine devices in 261 (85.3%), and oral contraceptive pills in 259 (84.6%).

Knowledge of male sterilisation was reported by 172 (56.2%) participants. Awareness of traditional methods such as withdrawal and rhythm methods was reported by 170 (55.6%) and 65 (21.2%) participants, respectively. Awareness of injectable contraception (DMPA/Antara) was reported by 164 (53.6%) participants, while 158 (51.6%) reported awareness of lactational amenorrhoea. Emergency contraceptive pills were known to 86 (28.1%) participants, and implants were the least known method, reported by 30 (9.8%).

With respect to community health worker engagement, 223 (72.9%) participants reported having had contact with an ASHA within the six months preceding the interview, while 83 (27.1%) reported no such contact during this period.

Method mix and current contraceptive use

Table 5 presents patterns of contraceptive use, method mix, partner support, and decision-making among eligible couples.

**Table 5:** Patterns of contraceptive use, method mix, partner support, and decision-making among eligible couples (N = 306). OCP: oral contraceptive pills; DMPA: depot medroxyprogesterone acetate, IUCD: intra uterine contraceptive device; IUD: intra uterine device.

Section	Category	n	%
Ever used any contraceptive method	Yes	207	67.6
	No	99	32.4
Currently using any contraceptive method	Yes	179	58.5
	No	127	41.5
Current method type (% of total N = 306)	Condoms	73	23.9
	Female sterilisation	56	18.3
	IUD/IUCD	18	5.9
	Pills (OCP)	17	5.6
	Injectables (DMPA/Antara)	14	4.6
	Withdrawal method	4	1.3
Partner support among current users (n = 179)	Yes	171	95.54
	Don't know	7	3.91
	No	1	0.55
Decision-making for current method (n = 179)	Joint decision (husband and wife)	96	53.63
	Husband	46	25.70
	Healthcare provider	21	11.73
	Self	16	8.94

Among the 306 eligible couples included in the study, 207 (67.6%) reported ever using any contraceptive method, while 179 (58.5%) were currently using contraception at the time of the survey (Table 5). Conversely, 99 (32.4%) participants reported never having used any contraceptive method, and 127 (41.5%) were not using any method currently.

With respect to the current contraceptive method mix, condoms were the most commonly reported method, used by 73 (23.9%) participants, followed by female sterilisation in 56 (18.3%). Use of modern spacing methods was less frequent, with intrauterine devices reported by 18 (5.9%), oral contraceptive pills by 17 (5.6%), and injectable contraception reported by 14 (4.6%) participants. Withdrawal was reported by 4 (1.3%) participants.

Among current contraceptive users (n = 179), partner support for contraceptive use was reported by 171 (95.5%) participants, while 7 (3.9%) reported uncertainty regarding partner support and 1 (0.6%) reported lack of support. Regarding decision-making for the current contraceptive method, joint decision-making by husband and wife was reported by 96 (53.6%) participants. Decisions made by the husband alone were reported by 46 (25.7%) participants, decisions initiated by healthcare providers were reported by 21 (11.7%), and self-initiated decisions by women were reported by 16 (8.9%).

Determinants of current contraceptive use

Bivariate analysis was performed to examine the association between current contraceptive use and selected independent variables. Associations between current contraceptive use and categorical variables, including age group, educational attainment, occupation, religion, monthly family income category, and contact with ASHA workers, were assessed using the chi-square test. Comparisons involving continuous variables, including age at marriage, duration of marriage, number of living children, number of living male children, number of living female children, and age of the youngest child, were conducted using the Wilcoxon rank-sum test, as these variables showed non-parametric distribution.

In bivariate analysis, current contraceptive use showed statistically significant associations with age group, education level, monthly family income, contact with ASHA workers, duration of marriage, number of living children, number of living male children, number of living female children, and age of the youngest child. No statistically significant association was observed with occupation, religion, or age at marriage.

Multivariable logistic regression analysis was performed to identify independent determinants of current contraceptive use. The fully adjusted model included age group, educational attainment, monthly household income, duration of marriage, number of living children, number of male children, age of youngest child, and contact with ASHA workers.

In the fully adjusted multivariable logistic regression model (Table 6), contact with ASHA workers was independently associated with current contraceptive use (AOR 3.48; 95% CI: 1.79-6.77; p < 0.001). Duration of marriage showed a statistically significant positive association, with a 14% increase in the odds of contraceptive use for each additional year of marriage (AOR 1.14; 95% CI: 1.02-1.28; p = 0.026). The number of male children was a strong independent determinant (AOR 3.17; 95% CI: 1.72-5.84; p < 0.001), indicating the continued influence of son preference on contraceptive behaviour.

**Table 6:** Fully adjusted multivariable logistic regression analysis of factors associated with current contraceptive use. ASHA: Accredited Social Health Activists.

Predictor	Adjusted odds ratio (AOR)	95% CI	p-value
Duration of marriage (years)	1.14	1.02–1.28	0.026
Number of living children	1.01	0.63–1.61	0.978
Number of male children	3.17	1.72–5.84	<0.001
Age of the youngest child (years)	0.99	0.85–1.14	0.850
Contact with ASHA (yes vs no)	3.48	1.79–6.77	<0.001
Age group (years)			
25–29 vs 18–24	0.70	0.32–1.55	0.380
30–34 vs 18–24	0.63	0.20–2.03	0.441
35–39 vs 18–24	0.36	0.07–2.04	0.251
≥40 vs 18–24	0.34	0.04–2.96	0.327
Education (ref: no schooling)			
Graduate and above	3.82	0.50–28.93	0.195
Higher secondary (10–12 years)	10.85	3.21–36.69	<0.001
Primary (1–5 years)	3.67	1.16–11.62	0.027
Secondary (6–10 years)	6.64	2.15–20.56	0.001
Monthly household income (ref: <15,000 INR)			
15,000–19,999	0.48	0.11–2.14	0.333
20,000–24,999	1.67	0.57–4.88	0.348
25,000–29,999	1.46	0.41–5.21	0.559
≥30,000	1.23	0.40–3.73	0.720

Educational attainment demonstrated a clear and graded association with contraceptive use. Compared to women with no schooling, women with primary education had higher odds of contraceptive use (AOR 3.67; 95% CI: 1.16-11.62; p = 0.027), those with secondary education had still higher odds (AOR 6.64; 95% CI: 2.15-20.56; p = 0.001), and women with higher secondary education showed the strongest association (AOR 10.85; 95% CI: 3.21-36.69; p < 0.001). Although women who were graduates or above also showed higher odds, this association was not statistically significant (AOR 3.82; p = 0.195), likely due to small numbers in this category.

In contrast, the total number of living children and the age of the youngest child were not independently associated with contraceptive use after adjustment. Age group and monthly household income were also not independently associated with contraceptive use after adjustment.

Recent contact with frontline health workers emerged as an important service-related factor influencing contraceptive use. Participants who reported contact with an ASHA within the preceding six months had significantly higher odds of current contraceptive use compared to those without such contact (AOR 3.48; 95% CI: 1.79-6.77). With respect to decision-making, contraceptive choice was most commonly reported as a joint decision between husband and wife (53.6%), followed by husband-led decisions (25.7%), while autonomous female decision-making was reported by a smaller proportion (8.9%).

From a life-course perspective, a substantial proportion of participants were young (33.0% aged 18-24 years) and childless (18.0%), indicating that non-use of contraception in these groups may reflect intentional fertility preferences rather than lack of access or awareness.

## Discussion

This community-based cross-sectional study provides comprehensive evidence on the prevalence, method mix, and determinants of contraceptive use among eligible couples in an urban resettlement colony of Delhi. The study found that 58.5% of eligible couples were currently using contraception, with condoms (23.9%) and female sterilisation (18.3%) being the predominant methods. Awareness of contraception was nearly universal (98.0%), but awareness and use of DMPA remained low at 53.6% and 4.6%, respectively. Contact with ASHA workers, educational attainment, duration of marriage, and number of male children emerged as independent predictors of current contraceptive use.

The current contraceptive prevalence of 58.5% observed in this study is lower than the urban Delhi CPR of 76.4% reported in NFHS-5 [4]. This disparity likely reflects the unique socioeconomic and healthcare access challenges faced by residents of urban resettlement colonies compared to the broader urban population. Similar findings have been reported in other urban slum studies in India, where CPR ranged from 45% to 65% [9-11]. The lower prevalence in resettlement colonies underscores the need for targeted interventions in these underserved urban settings.

The method mix observed in this study was dominated by condoms and female sterilisation, consistent with national trends [4]. However, the relatively higher use of condoms (23.9%) compared to female sterilisation (18.3%) in this urban resettlement setting contrasts with the national pattern, where female sterilisation remains the most prevalent method [4]. This finding may reflect greater access to condoms through public health programmes and pharmacies, as well as preferences for reversible methods among younger couples who have not yet completed their desired family size [12].

The low uptake of DMPA (4.6%) despite moderate awareness (53.6%) is a key finding of this study. This pattern suggests that awareness alone is insufficient to drive method adoption, and that barriers related to method-specific concerns, misinformation, and service delivery gaps play a critical role [13].

The strong association between ASHA contact and current contraceptive use (AOR 3.48) highlights the critical role of community health workers in facilitating access to family planning services. ASHAs serve as a bridge between the community and the formal health system, providing information, counselling, and referrals for contraceptive services [14]. This finding underscores the importance of strengthening ASHA training, supervision, and support to enhance their effectiveness in promoting contraceptive uptake, particularly for underutilised methods such as DMPA [15].

Educational attainment emerged as a strong and graded predictor of contraceptive use, with women who had higher secondary education showing more than ten times the odds of contraceptive use compared to women with no schooling (AOR 10.85). This finding is consistent with extensive literature demonstrating that education empowers women with knowledge, autonomy, and decision-making capacity, all of which facilitate contraceptive adoption [16,17]. Interventions aimed at improving female literacy and education in urban resettlement colonies may therefore have significant spillover benefits for reproductive health outcomes.

The persistence of son preference as a determinant of contraceptive use (AOR 3.17 for number of male children) is a concerning finding that reflects deeply entrenched gender norms in Indian society [18]. Couples with male children were significantly more likely to adopt contraception, suggesting that fertility decisions are influenced not only by the total number of children but also by their sex composition. Addressing son preference requires multifaceted interventions that challenge gender norms, promote gender equity, and strengthen implementation of laws prohibiting sex-selective practices [19].

Decision-making regarding contraceptive use was predominantly joint (53.63%), which is a positive indicator of spousal communication and shared reproductive decision-making. However, a substantial proportion of decisions were made by husbands alone (25.70%), highlighting the continued influence of patriarchal norms in reproductive health [20]. Interventions that engage men as partners in family planning and promote couple counselling may help to shift decision-making dynamics toward greater gender equity [21].

Partner support for contraceptive use was nearly universal (95.54%), which is encouraging and suggests that most husbands are supportive of their wifes’ contraceptive use. However, the high level of husband-led decision-making indicates that support does not necessarily translate into shared decision-making power. Programmes should aim to move beyond passive support toward active engagement of men in reproductive health decision-making [22].

The findings of this study indicate that contraceptive use among eligible couples in this urban resettlement colony is influenced by life-course factors, method mix, and health system contact rather than awareness alone. Although awareness of contraception was nearly universal, current use was substantially lower, reflecting a skewed method mix characterised by predominance of condoms and comparatively low uptake of other modern spacing methods such as intrauterine devices, oral contraceptive pills, and injectable contraception. Importantly, lower contraceptive use among younger and childless couples should be interpreted cautiously, as a substantial proportion of participants were in early stages of marital life, and non-use in these groups may reflect intentional fertility preferences rather than unmet need. Service-related factors emerged as significant, with recent contact with ASHA showing a strong independent association with current contraceptive use, underscoring the role of community-based family planning services in facilitating uptake. Decision-making patterns further revealed that contraceptive choices were predominantly joint or husband-led, with limited autonomous female decision-making, indicating potential gaps in equitable participation. Overall, these findings suggest that strengthening community-based family planning services in urban resettlement settings should prioritise improved counselling, informed method choice, and more inclusive decision-making processes, while accounting for fertility intentions across the reproductive life course.

This study provides community-based evidence on contraceptive use from an urban resettlement colony, a population that is frequently underrepresented in routine health statistics and national surveys. The use of simple random sampling of households enhanced internal validity and minimised selection bias. Data were collected using a pre-tested questionnaire adapted from the National Family Health Survey, enabling comparability with state and national estimates. Beyond estimating overall contraceptive prevalence, the study examined method mix and a range of socio-demographic and programmatic factors, offering a more detailed understanding of contraceptive use patterns in an urban resettlement context. The application of multivariable logistic regression further strengthened the analysis by identifying factors independently associated with current contraceptive use.

The findings of this study should be interpreted in light of several limitations. First, the cross-sectional design precludes causal inference, and observed associations between participant characteristics, service-related factors, and contraceptive use cannot be interpreted as temporal or causal relationships. Second, contraceptive use and related variables were self-reported and may therefore be subject to recall bias or social desirability bias, particularly for sensitive aspects of reproductive behaviour. Importantly, the study did not directly measure unmet need for family planning, current fertility intentions, or pregnancy desire, which limits the interpretation of contraceptive non-use. A proportion of couples in the study were young or childless, and non-use of contraception among these groups may reflect intentional desire for pregnancy rather than low acceptance or unmet need. Although the data collection tool included items on reasons for non-use of contraception, these variables were not analysed in the present study, limiting the ability to distinguish between voluntary non-use and non-use related to access, method-related concerns, or partner dynamics.

While the study assessed selected socio-demographic, reproductive, and service-contact factors, other potentially important determinants of contraceptive behaviour, such as psychosocial influences, couple communication, cultural norms, and perceptions of specific contraceptive methods, were not comprehensively examined. In addition, health system factors were measured primarily through indicators of service contact rather than detailed facility-level or supply-side assessments.

Finally, the study was conducted in a single urban resettlement colony, which may limit the generalisability of the findings to other urban settings or to rural populations with different social and health system contexts. Despite these limitations, the study provides a detailed description of contraceptive use patterns and associated factors in an under-studied urban resettlement population.

## Conclusions

The study provides evidence that contraceptive use among eligible couples in an urban resettlement colony is not primarily constrained by lack of awareness, but varies according to stage in the reproductive life course, contraceptive method mix, and engagement with community-level health services. Patterns observed in the analysis indicate that contraceptive adoption increases with progression of marital life and childbearing, while method choice remains concentrated around a limited set of options, particularly condoms and permanent methods.

The consistent association between recent contact with frontline health workers and current contraceptive use highlights the role of community-based service contact in shaping utilisation within this setting. At the same time, decision-making around contraception remains largely shared or male-influenced, suggesting that contraceptive behaviour is embedded within household dynamics rather than individual choice alone. Overall, the findings underscore the need to interpret contraceptive non-use within its demographic and social context and provide a descriptive basis for understanding contraceptive patterns in urban resettlement populations.

## Appendices

Appendix A 

**Table 7:** Questionnaire. Adapted from the National Family Health Survey-5 (NFHS-5) [4], International Institute for Population Sciences (IIPS) and ICF. The NFHS-5 questionnaire is publicly available and was adapted for academic research purposes. ANMs: auxiliary nurse midwives; DMPA: depot medroxyprogesterone acetate; IEC: Information, Education, and Communication; OCP: oral contraceptive pills; DMPA: depot medroxyprogesterone acetate; IUCD: intra uterine contraceptive device; IUD: intra uterine device; PHC: Primary Health Centre; USHA: Urban Slum Health Action Programme.

Section	Question number	Question/item	Response options/measurement
A. Socio-demographic details	A1	Name	Open-ended
	A2	Age (in completed years)	Numeric
	A3	House number	Open-ended
	A4	Education	No schooling; Primary (1–5); Secondary (6–10); Higher secondary (11–12); Graduate and above
	A5	Occupation	Not working; Petty business; Domestic worker; Private employee; Government employee
	A6	Monthly household income (INR)	Numeric/Categories
	A7	Religion	Hindu; Muslim; Christian; Sikh; Jain; Buddhist
	A8	Caste	Open-ended
B. Marital status and family structure	B1	Age at marriage (years)	Numeric
	B2	Duration of marriage (years)	Numeric
	B3	Type of family	Nuclear; Joint; Extended
C. Reproductive history	C1	Number of living children	Numeric
	C2	Number of male children	Numeric
	C3	Number of female children	Numeric
	C4	Age of youngest child (years)	Numeric
	C5	Desired family size	Numeric
D. Contraceptive awareness and practices	D1	Ever heard of family planning methods	Yes; No
	D2	Methods known	OCP; Condoms; IUCD; Injectables (DMPA/Antara); Implants; Female sterilisation; Male sterilisation; ECP; LAM; Rhythm; Withdrawal
	D3	Source of information	Health facility IEC; ASHA/USHA; TV; Newspaper; Radio; Friends; Relatives
	D4	Ever used any contraceptive method	Yes; No
	D5	Methods ever used	Same as D2
	D6	Duration of use (months)	Numeric
	D7	Source of previously used method	Government facility; Private facility; ASHA/USHA; Pharmacy
	D8	Currently using any method	Yes; No
	D9	Current method used	Single response from D2
	D10	Side effects from current method	Yes; No; Description
	D11	Source of current method	Government facility; Private facility; ASHA/USHA; Pharmacy
	D12	Decision maker	Self; Husband; Joint; Health care provider
	D13	Partner support	Yes; No; Do not know
E. DMPA-specific knowledge and determinants	E1	Heard of injectable contraceptives (DMPA/Antara/Sayana Press)	Yes; No
	E2	Ever used injectable contraceptives	Yes; No
	E3	Currently using DMPA	Yes; No
	E4	Duration of DMPA use if discontinued (months)	Numeric
	E5	Reasons for discontinuation	Side effects; Desire for pregnancy; Access; Cost; Partner objection
	E6	Reasons for choosing DMPA	Long acting; Convenient; Privacy; Effectiveness; Partner preference; No daily action; Provider recommendation
	E7	Influencer for DMPA use	Self; Husband; Friends/relatives; ASHA/USHA; ANM
	E8	Partner support for DMPA	Yes; No; Do not know
	E9	Place of DMPA injection	PHC; Sub-centre; Private clinic; Home visit
	E10	Mode of travel	Walking; Own vehicle; Public transport
	E11	Financial assistance for DMPA	Yes; No
F. Side effects and counselling	F1	Experienced side effects with DMPA	Yes; No
	F2	Type of side effects	Irregular bleeding; Heavy bleeding; Amenorrhea; Weight gain/loss; Headache; Mood changes; Decreased libido
	F3	Severity of side effects	Mild; Moderate; Severe; Very severe
	F4	Time for side effects to subside (months)	Numeric
	F5	Received counselling on DMPA	Yes; No
	F6	Informed about side effects	Yes; No
	F7	Informed about other methods	Yes; No
	F8	Satisfaction with counselling	Very satisfied; Satisfied; Neutral; Dissatisfied; Very dissatisfied
G. Future contraceptive intentions	G1	Desire for more children	Yes; No
	G2	Future contraceptive intention	Yes; No; Planning pregnancy
	G3	Preferred future method	Condoms; IUCD; Injectables; Pills; Tubectomy; Vasectomy; Implants; Natural methods
	G4	Reason for not choosing DMPA	Lack of awareness; Safety concerns; Weight gain; Pain; Bleeding; Amenorrhea; Reduced sexual pleasure; Better methods
H. Open-ended questions	H1	Facilitators for contraceptive use	Open-ended
	H2	Barriers to contraceptive use	Open-ended
	H3	Advantages of DMPA	Open-ended
	H4	Disadvantages of DMPA	Open-ended
I. ASHA contact	I1	Contact with ASHA in last six months	Yes; No
